# Evaluation of inverted papilloma recurrence rates and factors associated recurrence after endoscopic surgical resection: a retrospective review

**DOI:** 10.1186/s40463-023-00638-5

**Published:** 2023-04-27

**Authors:** Sheila Yu, Elysia Grose, Daniel J. Lee, Vincent Wu, Mitchell Pellarin, John M. Lee

**Affiliations:** 1 Division of Rhinology, Department of Otolaryngology – Head and Neck Surgery, St. Michael’s Hospital, Unity Health Toronto, University of Toronto, 30 Bond Street, 8 Cardinal Carter Wing, Toronto, ON M5B 1W8 Canada; 2 Department of Otorhinolaryngology-Head and Neck Surgery, Perelman School of Medicine, University of Pennsylvania, Philadelphia, PA USA; 3 Faculty of Health Sciences, McMaster University, Hamilton, ON Canada

**Keywords:** Inverted papilloma, Sinonasal tumour, Endoscopic sinus surgery, Recurrence, SNOT-22

## Abstract

**Background:**

Sinonasal inverted papillomas (IP) are benign tumours arising from the mucosal lining of the nasal cavity and paranasal sinuses with a high propensity for recurrence and malignant transformation. Advances in endoscopic surgery and improved radiologic navigation have increased the role of endoscopic surgical resection in the treatment of IPs. The current study aims to evaluate the rate of IP recurrence after endoscopic endonasal resection and to evaluate factors which impact recurrence.

**Methods:**

This was a single-centre retrospective chart review of all patients who underwent endoscopic sinus surgery for management of IP between January 2009 and February 2022. Primary outcomes were the rate of IP recurrence and time to IP recurrence. Secondary outcome measures were patient and tumour factors that contributed to IP recurrence.

**Results:**

Eighty-five patients were included. The mean age was 55.7 and 36.5% of patients were female. The mean follow-up time was 39.5 months. Of the 85 cases, 13 cases (15.3%) had recurrence of their IP and the median time to recurrence was 22.0 months. All recurrent tumours recurred at the attachment site of the primary tumour. The univariate analysis did not identify any significant demographic, clinical, or surgical predictors of IP recurrence. There were no significant changes in sinonasal symptoms at the time IP recurrence was detected.

**Conclusion:**

Endoscopic endonasal resection of IPs represents an effective surgical approach, however, the relatively high rate of recurrence and lack of symptomatic changes at the time of recurrence necessitates long term follow up. Better delineation of risk factors for recurrence can help identify high-risk patients and inform postoperative follow up strategies.

## Introduction

Inverted papillomas (IPs) are benign tumours arising from the Schneiderian mucosa of the nasal cavity and paranasal sinuses with either single or multifocal sites of origin [1]. Clinically, patients with IPs present with unilateral sinonasal symptoms such as nasal obstruction and are classically found to have “unilateral nasal polyps” on nasal endoscopy [2–4]. Although IPs are benign tumours, the malignant transformation rate ranges from 5 to 15%, necessitating complete surgical resection and continued surveillance [5–7]. With the advances in endoscopic technology and surgical skills, the mainstay of surgical resection has become a complete endoscopic endonasal resection with identification of the attachment point(s). Open or combined approach is often reserved for complex tumours with extensive frontal sinus involvement, anterolateral and/or inferior maxillary sinus lesions and cases of carcinoma transformation or extra-sinus extension [8–11]. However, the surgical management of IP is challenging with a high recurrence rate ranging from 13 to 35% [12–15]. Notable factors that may increase the chance of recurrence are the multifocality of the tumour attachment and prior surgery [8, 16–18]. The objective of this study is to evaluate the clinical outcomes of endoscopic IP resection and assess clinical factors that may impact the recurrence at a high-volume Canadian tertiary centre.

## Methods

### Study design and patient population

Approval for this study was granted by the Unity Health Toronto Research Ethics Board (REB #20-012) at St. Michael’s Hospital, Toronto, Ontario, Canada. A retrospective review of all adult patients with a diagnosis of IP and who underwent endoscopic resection between January 2009 and February 2022 at St. Michael’s Hospital was conducted. All endoscopic endonasal resections were performed by the study’s senior surgeon (JML), a fellowship trained rhinologist. Patients were included if they were 18 years of age or older and had a histopathologically confirmed inverted papilloma. Patients who were under 18 years of age and patients who had a diagnosis of squamous cell carcinoma at the time of the initial consultation were excluded from this study.

All patients underwent computed tomography (CT) scans of the sinuses preoperatively. Contrast-enhanced MRI was completed when there were areas of skull base, orbital or carotid artery dehiscence around the suspected tumour site. Additionally, MRI was ordered for all suspected frontal sinus and sphenoid sinus involvement to help ascertain the extent of disease and site of attachment for surgical planning. All patients were seen at regular postoperative visits at 1 week, 1 month, 3 months, 6 months, 1 year, and annually thereafter unless closer follow-up was clinically necessary. Patients completed a 22-item Sino-Nasal Outcome Test (SNOT-22) at each follow-up visit.

### Surgical approach and follow up protocol

The general surgical approach has been described previously [19]. Intraoperative frozen sections were used to confirm a pathological diagnosis of IP. The IP was debulked with a microdebrider leaving the attachment site intact. The goal of the surgery was to identify attachment sites and remove the mucosa and underlying periosteum to decrease the chance of recurrence. Angled endoscopes, curved instruments, microdebriders, bipolar cautery and burrs were used for tumour resection and the attachment site ablation. Standard resection included maxillary antrostomy, ethmoidectomy, sphenoidotomy, and frontal sinusotomies. The site of attachment was drilled drown and cauterized with bipolar cautery. Where possible, the bone near the site of attachment was completely removed. Medial maxillectomy, sphenoid drill out and frontal sinus drill out (Draf III) were employed for diseases with attachment sites at the medial maxillary sinus wall, sphenoid sinus, or frontal sinus, respectively [11]. Postoperatively patients are seen every 3 months for the first year and then every 6 months for the next two years. After 3 years, patients are seen annually or sooner if they have symptoms [20]. Patients were typically followed for a lifetime.

### Outcome measurements and data collection

Baseline characteristics were collected and included age, gender, and disease presentation status (primary or residual/revision). Primary tumour attachment site(s) was defined as the attachment site(s) identified on initial endoscopic examination in the operating room at the time of first surgery at our institution, although CT images was used preoperatively to gauge the site of attachment. Similarly, Krouse classification was done retrospectively through review of the operative notes [21]. In addition, surgical technique(s), time to recurrence, and follow-up time were extracted. The SNOT-22 score was collected from follow-up notes in order to characterize the association between the severity of IP symptoms both prior and after endoscopic endonasal resection.

Primary outcomes were the rate of IP recurrence and time to IP recurrence. Secondary outcome measures were patient and tumour factors that contributed to IP recurrence.

### Statistical analysis

Descriptive statistics were used to summarise continuous and categorical variables. Differences in baseline characteristics between groups were compared using the Chi-squared or independent samples t-test for means, and the Mann–Whitney U test for medians.

Time to recurrence was calculated as the time between the first surgery and the date in which recurrence was identified. Evaluation of recurrence and factors contributing to recurrence of IP was performed with Kaplan–Meier methodology and Cox proportional hazards model.

All available preoperative and postoperative SNOT-22 scores were included in the central tendency calculation. Only patients who had documentation of at least one preoperative and one postoperative SNOT-22 score were included in the calculation of postoperative SNOT-22 changes. Preoperative and postoperative SNOT-22 scores were compared using the Wilcoxon signed rank test. All analyses were performed using SPSS software (version 28.0; SPSS Inc, Chicago, IL, USA), with two-sided statistical significance set to p < 0.05.

## Results

### Baseline patient characteristics

Eighty-five patients with IP were included. Patient demographic characteristics based on presence of recurrence are shown in Table 1. The mean age at the time of surgery was 55.7 years (SD: 14.8). Thirty-seven patients (43.5%) had prior endoscopic resection at other institutions. The mean follow up time was 36.4 months (SD: 33.8), with twenty-six patients (30.6%) followed for 12 months or less, 16 patients (18.8%) for 12 to 24 months, five patients (5.9%) for 24 to 36 months, five patients (5.9%) for 36 to 48 months, and 33 patients (38.8%) for 48 months or more. Clinical characteristics and extent of surgery performed in this patient cohort are shown in Table 2. There were no differences in baseline characteristics between recurrent and non-recurrent groups. There were two cases of malignant transformation to squamous cell carcinoma in this cohort. Neither of the two cases who had malignant transformation had known allergies, had a history of smoking, or had a history of HPV. Furthermore, neither patient had any evidence of dysplasia on initial pathology.

**Table 1:** Demographic characteristics

	Total cohort (n = 85)	IP recurrence (15.3%, n = 13)	No IP recurrence (85.7%, n = 72)	HR	95% CI	*p* value
Age at surgery, in years Mean (SD)	55.7 (14.8)	56.5 (7.0)	55.6 (15.9)	1.02	0.98–1.06	0.45
Sex Female, n (%)	31 (36.5)	3 (23.1)	28 (38.9)	0.40	0.11–1.50	0.17
Follow-up time, in months Mean (SD)	36.4 (33.8)	57.1 (31.4)	39.5 (34.1)	0.97	0.95–1.00	0.07
Prior surgery at external site, n (%)	37 (43.5)	6 (46.2)	31 (43.1)	1.28	0.41–4.03	0.67

*CI* confidence interval; *HR* hazard ratio; *IP* inverted papilloma; *IQR* interquartile range; *SD* standard deviation

**Table 2:** Clinical and surgical characteristics of patient cohort

	Total cohort (n = 85)	IP recurrence (15.3%, n = 13)	No IP recurrence (85.7%, n = 72)	HR	95% CI	*p* value
Type of surgery performed*, n (%)						
Standard Endoscopic resection**	29 (34.1)	26 (36)	3 (23)	1.04	0.28–3.86	0.95
Extended resection†	56 (65.8)	46 (64)	10 (77)	0.96	0.26–3.57	0.95
*IP attachment site*, n (%)						
Maxillary	43 (50.6)	6 (46.2)	37 (51.4)	0.79	0.25–2.47	0.69
Ethmoid	22 (25.9)	3 (23.1)	19 (26.4)	0.50	0.11–2.28	0.37
Frontal sinus	9 (10.6)	1 (7.7)	8 (11.1)	0.94	0.11–7.31	0.95
Sphenoid	6 (7.1)	1 (7.7)	5 (6.9)	0.91	0.12–7.07	0.93
Skull base	16 (18.8)	3 (23.1)	13 (18.1)	0.66	0.15–3.04	0.60
Extra-sinus§	44 (51.8)	6 (46.2)	38 (52.8)	1.28	0.41–4.02	0.67
*Maxillary sinus involvement*, n (%)						
Posterior wall	16 (18.8)	2 (15.4)	14 (19.4)	0.70	0.15–3.20	0.65
Inferior wall	14 (16.5)	2 (15.4)	12 (16.7)	1.04	0.23–4.75	0.96
Lateral wall	21 (24.7)	5 (38.5)	16 (22.2)	1.73	0.55–5.44	0.35
Anterior wall	17 (20.2)	4 (30.8)	13 (18.3)	1.70	0.51–5.68	0.39
Medial wall	23 (27.1)	3 (23.1)	20 (27.8)	0.89	0.24–3.29	0.86
Superior wall	11 (12.9)	2 (15.4)	9 (12.5)	1.07	0.23–4.90	0.94
Single attachment site, n (%)	30 (35.3)	4 (30.8)	26 (36.1)	0.70	0.19–2.60	0.60
Multiple attachment sites, n (%)	54 (64.3)	9 (69.2)	45 (62.5)	1.43	0.39–5.27	0.60
Krouse, median (IQR)	3 (2–3)	3 (2–3)	3 (2–3)	1.23	0.52–2.92	0.63

*CI* confidence interval; *HR* hazard ratio; *IP* inverted papilloma; *IQR* interquartile range

*This refers to the initial surgery performed at St. Michael’s Hospital

**Maxillary antrostomy, ethmoidectomy, sphenoidotomy, frontal sinusotomy (Draf 2A, 2B)

†Includes extended sphenoid drill out, Draf3, or modified medial maxillectomy in combination with standard endoscopic resection

§Includes nasal floor, lateral nasal wall, uncinate process, inferior turbinate, middle turbinate, superior turbinate, nasal septum, lamina papyracea, and frontal recess

### Recurrence free survival analysis

**Fig. 1:**
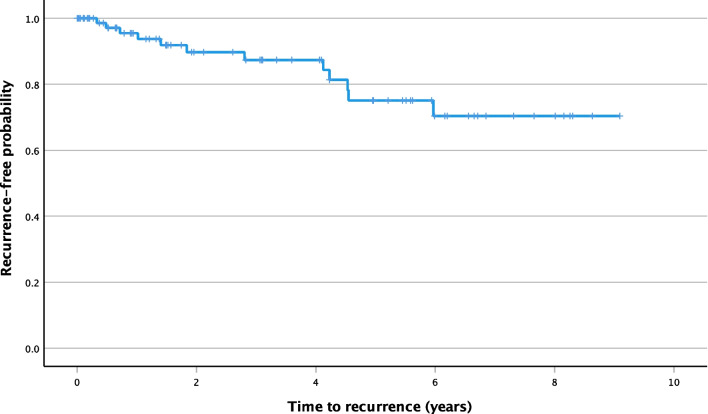
Kaplan–Meier curve for time to recurrence for patients with inverted papilloma

Of the 85 patients who underwent endoscopic endonasal resection, the overall recurrence rate was 15.3% (13/85). The median time to recurrence was 22.0 months (IQR: 10.4 to 52.6). The probability of remaining free of recurrence at 1, 3, and 5 years was 95.5% (SD: 2.6%), 87.4% (SD: 4.6%), and 75.1% (SD: 6.9%), respectively (Fig. 1).

### Recurrence and primary tumour location

The attachment sites of the primary IPs are shown in Table 3. On recurrence, all recurrent tumours were confirmed on pathology to be IP, and all involved the site of the primary tumour. Four of the 13 recurrences developed in patients who had a single site of attachment (n = 30 in total cohort) at the time of IP diagnosis and 9 recurrent cases had a multi-origin attachment site (n = 54 in total cohort) at the time of initial diagnosis. The site of origin of IP did not show significant influence on the frequency of disease recurrence. Univariate Cox regression analysis did not identify any significant tumour or surgical factors as predictors of IP recurrence (Tables 1 and 2).

**Table 3:** Attachment of primary tumour

Primary tumour attachment site(s)*	Total cohort (n = 85)	IP recurrence (15.3%, n = 13)	No IP recurrence (85.7%, n = 72)
Maxillary (%)	43 (50.6)	6 (46.2)	37 (51.4)
Ethmoid (%)	22 (25.9)	3 (23.1)	19 (26.4)
Frontal sinus (%)	9 (10.6)	1 (7.7)	8 (11.1)
Sphenoid (%)	6 (7.1)	1 (7.7)	5 (6.9)
Skull base (%)	16 (18.8)	3 (23.1)	13 (18.1)
Extra-sinus (%)**	44 (51.8)	6 (46.2)	38 (52.8)

*IP* inverted papilloma

*Primary tumour attachment site(s) was defined as the attachment sites identified on initial endoscopic examination in the operating room at the time of first surgery at our institution

**Includes nasal floor, lateral nasal wall, uncinate process, inferior turbinate, middle turbinate, superior turbinate, nasal septum, lamina papyracea, and frontal recess

### Postoperative SNOT-22 outcomes following endoscopic endonasal resection

In the overall cohort, there was a significant decrease in median postoperative SNOT-22 score when compared to preoperative SNOT-22 scores of 13.0 points (IQR: − 40.0 to − 3.5; *p* = 0.001) at 3 months, 18.3 points (IQR: − 37.7 to − 7.0; *p* < 0.001) at 6 months, and 12.0 points (IQR: − 36.0 to − 4.0; *p* = 0.002) at one year.

**Fig. 2:**
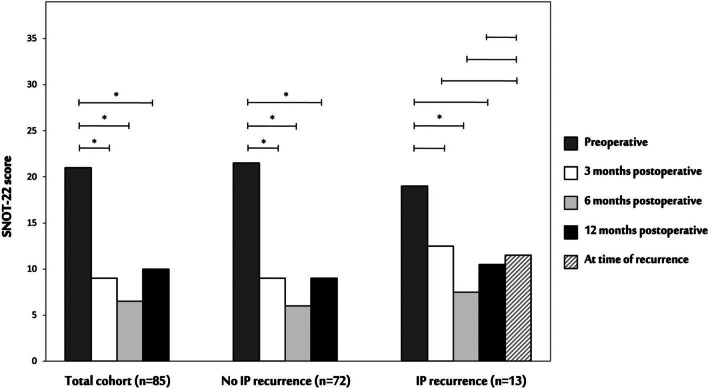
Postoperative changes in SNOT-22 scores. The dark grey bars represent preoperative SNOT-22 scores. The white bars represent the SNOT-22 scores at 3 months postoperatively. The light grey bars represent the SNOT-22 scores at 6 months postoperatively. The black bars represent the SNOT-22 scores at 12 months postoperatively. The dashed bar represents the SNOT-22 score at the time of IP recurrence. *p < 0.05*.* IP = inverted papilloma; SNOT-22 = 22-item Sino-Nasal Outcome Test

Figure 2 illustrates the median preoperative and postoperative SNOT-22 scores. There was no difference in SNOT-22 scores between the recurrence and the non-recurrence groups both preoperatively and at 1, 3 and 6 months postoperatively. Among the recurrence cohort, there was no significant increase in the SNOT-22 scores at the time when recurrence was detected when compared to postoperative scores.

## Discussion

It is well established that sinonasal IPs have a high propensity for recurrence, with cited rates of 13–35% [9, 13–15]. Endoscopic surgical management of IPs has become the cornerstone of treatment for these patients due to advances in endoscopic tools and techniques. In fact, the endoscopic approach offers comparable outcomes with less morbidity when compared to external approaches [22]. Thus, the aim of the current retrospective review was to further evaluate recurrence rates, factors associated with recurrence, and quality of life outcomes for patients with IP who underwent endoscopic management at a high-volume Canadian tertiary care centre.

In this study, 15.3% of cases were found to have recurrent disease during a mean follow-up time of 36.4 months, with the median time to recurrence being 22.0 months. A recent meta-analysis of 23 studies including a total of 2451 patients reported a recurrence rate ranging from 1 to 30% for patients undergoing endoscopic resection of IPs during a follow-up time ranging from 14 to 55.5 months which demonstrates the heterogeneity in the existing literature regarding the recurrence rates of IPs [13]. Multiple factors can influence the reported recurrence rates in these studies including the length of follow up time, surgical technique, and the different types of IPs [19, 22, 23]. Our study found that the probability of remaining free of recurrence decreases with increasing length of follow-up. This finding is concordant with the existing literature which reports greater rates of recurrence beyond 3–5 years of follow up, with late recurrences occurring up to 15 years after primary resection [24–26]. The patient population in the current study is unique in that it included all locations of IPs in addition to a large proportion of patients (43.5%) who had prior sinus surgery, which is well documented as a risk factor for recurrent disease [8, 18]. This association is thought to result from incomplete resection, distortion of anatomy from previous surgery, scarring, or spread of disease to other locations in the case of misdiagnosed IP for nasal polyps [27, 28]. Thus, the results of this study may be more representative of the recurrence rates expected in a tertiary care referral centre given the large number of patients who have undergone previous surgeries.

Although there is a large body of literature describing risk factors for inverted papilloma recurrence, the results of these studies are conflicting and still debated [14]. In this study, site of tumour attachment and having multiple attachment sites were not associated with a higher rate of recurrence. Attachment of IP to the frontal sinus is a commonly reported association with increased recurrence rates of IP due to the technical difficulty of accessing the frontal sinus, limited visualisation via endoscopy, and difficulty obtaining a margin when up against critical structures [29]. Statistically significant recurrence rates up to 27.3% have been reported [16, 17, 29]. In contrast, our study found only 7.7% of recurrent cases to have had a primary tumour attachment site at the frontal sinus compared with non-recurrent cases which had a frontal sinus origin of 11.1%. This may be due to the usage of advanced frontal sinus techniques, such as Draf III procedures. In fact, there is emerging evidence that Draf III procedures in the context of IPs based in the frontal sinus or recess may reduce recurrence rates in addition to preventing external scarring and frontal outflow tract obstruction [30]. Similarly, maxillary sinus origin of IPs also have a reported association with increased IP recurrence due to more challenging visualisation and access of the anterior, medial and inferior maxillary sinus walls via transnasal endoscopic approach [17, 31, 32]. Although our study had a substantial amount of anterior, medial and inferior maxillary wall involvement (30.8%, 23.1% and 15.4%, respectively) amongst recurrent cases, we did not observe a significant association with IP recurrence and the location of maxillary sinus wall. Furthermore, in this study, 69% of patients who had recurrence and 63% of patients with no recurrence had multiple sites of attachment. Having multiple sites of attachment was not significantly associated with recurrence. In contrast, other large studies have found that having multiple sites of tumour attachment is significantly associated with recurrence [33]. The association between recurrence and multifocal disease is an area which certainly deserves further investigation as it is possible that complete resection would be made more challenging when multiple sites of attachment exist.

To further evaluate factors associated with recurrence, the IP tumour staging system proposed by Krouse et al. [5] was selected for statistical analysis in this study. Existing studies have shown inconsistent evidence on whether increasing Krouse tumour staging is associated with IP recurrence [12, 34–36]. Similarly, in the current study, increased Krouse stage was not found to be associated with recurrence. The Krouse staging system is determined by the extent of tumour periphery, which is less clinically meaningful than staging that the tumour attachment [14]. Tumours involving different paranasal sinuses may also be grouped into the same stage when they have variable associations with recurrence [8, 16–19, 31].

There is limited literature on the quality of life outcomes for patients undergoing endoscopic removal of IPs as many studies group all sinonasal and skull base tumours [37, 38]. van Samkar and Georgalas performed a study on the long-term quality of life outcomes in patients who underwent endoscopic removal of IP [39]. In a population of 27 patients, they found a postoperative SNOT-22 of 12.0 points during a median follow-up of 6 years, which was comparable with the average of 9.3 points reported by healthy people on SNOT-22 [39, 40]. Similarly, Bertazzoni et al. reported a mean postoperative SNOT-22 score of 5.3 points during a mean follow-up period of 66.3 months in 59 patients who had extended endoscopic maxillectomy for IP [41]. While both studies cite favourable postoperative SNOT-22 scores following endoscopic endonasal resection, no comparison was made with baseline preoperative SNOT-22 [39, 41]. More recently, Lin et al. reported no statistically significant improvements in SNOT-22 scores after endoscopic prelacrimal recess approach in 21 patients, although a downtrend was seen [42]. Our study found a statistically significant median decrease in SNOT-22 of 13 points at 3 months, 18 points at 6 months, and 12 points at one year. This demonstrates that undergoing endoscopic resection of IP does not predispose patients to subsequent symptoms of chronic rhinosinusitis and has benefit with respect to disease specific quality of life. Furthermore, this study demonstrated that there was no increase in SNOT-22 scores at the time of recurrence when compared to SNOT-22 scores after their initial resection. This was further validated by the fact that only 3 of the patients who recurred re-presented with sinonasal symptoms. To our knowledge, this is one of the first papers to investigate the change in sinonasal symptoms at the time IP recurrence is detected. The lack of change in symptoms in the majority of patients prior to recurrence further necessitates the need for long term follow up and serial endoscopic clinical examination to monitor for recurrence.

### Limitations

This study has several limitations. Firstly, it is a retrospective, single-centre study which limits the generalizability of the results. In addition, 37 patients in this cohort had undergone endoscopic endonasal resection at other institutions prior to receiving care at our institution, which could have impacted surgical difficulty, rate of recurrence, and length of time to recurrence. Although we aimed to capture long-term data on IP recurrence within our cohort and had a mean follow-up of 36.4 months, this may not have adequately captured all cases of recurrence, as literature has suggested follow-up of at least 5 years [43]. Furthermore, there is a large degree of variability in our follow up period given that 33% of our patient population was operated on from 2017 and later. However, there was only one patient in our study who was lost to follow up. Finally, the SNOT-22 provides a relatively comprehensive assessment of sinonasal outcomes in chronic rhinosinusitis patients but is not currently validated in the IP population despite wide use in rhinologic literature [44]. The potential that this instrument fails to completely capture important and relevant symptomatology related to IPs must be considered.

## Conclusion

In patients with IP who have undergone endoscopic surgical resection, this study found a recurrence rate of 15.3% with a median time to recurrence of 22.0 months. No demographic, surgical, or tumour variables were significantly associated with IP recurrence. These results suggest that endoscopic endonasal resection of IPs is an effective surgical approach despite potential surgical challenges associated with visualisation and access. However, the propensity for IPs to recur necessitates long-term follow up. To our knowledge, this is one of the largest single centre, single surgeon studies within recent years [14, 31, 34]. Completion of all surgeries at one institution by a single experienced senior surgeon reduces heterogeneity that may arise from varying surgical technique and institutional protocols to better elucidate the natural history of IP recurrence.

## Data Availability

The datasets generated and analyzed during the current study are available from the corresponding author on reasonable request.

## Abbreviations

CI: Confidence interval
HR: Hazard ratio
IP: Inverted papilloma
IQR: Interquartile range
SD: Standard deviation
SNOT-22: 22-Item Sino-Nasal Outcome Test

## References

[1] Wood JW, Casiano RR (2012). Inverted papillomas and benign nonneoplastic lesions of the nasal cavity. Am J Rhinol Allergy.

[2] Chaudhry IA, Taiba K, Al-Sadhan Y, Riley FC (2005). Inverted papilloma invading the orbit through the nasolacrimal duct: a case report. Orbit.

[3] Elner VM, Burnstine MA, Goodman ML, Dortzbach RK (1995). Inverted papillomas that invade the orbit. Arch Ophthalmol.

[4] Pitak-Arnnop P, Bertolini J, Dhanuthai K, Hendricks J, Hemprich A, Pausch NC (2012). Intracranial extension of Schneiderian inverted papilloma: a case report and literature review. Ger Med Sci.

[5] Krouse JH (2001). Endoscopic treatment of inverted papilloma: safety and efficacy. Am J Otolaryngol.

[6] Hyams VJ (1971). Papillomas of the nasal cavity and paranasal sinuses. A clinicopathological study of 315 cases. Ann Otol Rhinol Laryngol.

[7] Long C, Jabarin B, Harvey A (2020). Clinical evidence based review and systematic scientific review in the identification of malignant transformation of inverted papilloma. J Otolaryngol Head Neck Surg.

[8] Sciarretta V, Fernandez IJ, Farneti P, Pasquini E (2014). Endoscopic and combined external–transnasal endoscopic approach for the treatment of inverted papilloma: analysis of 110 cases. Eur Arch Otorhinolaryngol.

[9] Bugter O, Monserez DA, van Zijl FVWJ, de Jong RJB, Hardillo JA (2017). Surgical management of inverted papilloma; a single-center analysis of 247 patients with long follow-up. J Otolaryngol Head Neck Surg.

[10] Wassef SN, Batra PS, Barnett S (2012). Skull base inverted papilloma: a comprehensive review. ISRN Surg.

[11] Wang Y, An Y, Zhao C, Dong R, Cheng F (2020). Attachment-oriented endoscopic treatment of inverted papilloma involving the frontal sinus/recess. J Craniofac Surg.

[12] Kim JS, Kwon SH (2017). Recurrence of sinonasal inverted papilloma following surgical approach: a meta-analysis. Laryngoscope.

[13] Goudakos JK, Blioskas S, Nikolaou A, Vlachtsis K, Karkos P, Markou KD (2018). Endoscopic resection of sinonasal inverted papilloma: systematic review and meta-analysis. Am J Rhinol Allergy.

[14] Lee JJ, Roland LT, Licata JJ (2020). Morphologic, intraoperative, and histologic risk factors for sinonasal inverted papilloma recurrence. Laryngoscope.

[15] Nygren A, Kiss K, von Buchwald C, Bilde A (2016). Rate of recurrence and malignant transformation in 88 cases with inverted papilloma between 1998–2008. Acta Otolaryngol.

[16] Katori H, Nozawa A, Tsukuda M (2006). Histopathological parameters of recurrence and malignant transformation in sinonasal inverted papilloma. Acta Otolaryngol.

[17] Kim DY, Hong SL, Lee CH (2012). Inverted papilloma of the nasal cavity and paranasal sinuses: a Korean multicenter study. Laryngoscope.

[18] Lisan Q, Laccourreye O, Bonfils P (2017). Sinonasal inverted papilloma: risk factors for local recurrence after surgical resection. Ann Otol Rhinol Laryngol.

[19] Wu V, Siu J, Yip J, Lee JM (2018). Endoscopic management of maxillary sinus inverted papilloma attachment sites to minimize disease recurrence. J Otolaryngol Head Neck Surg.

[20] Lisan Q, Laccourreye O, Bonfils P (2016). Sinonasal inverted papilloma: FROM diagnosis to treatment. Eur Ann Otorhinolaryngol Head Neck Dis.

[21] Krouse JH (2000). Development of a staging system for inverted papilloma. Laryngoscope.

[22] Peng R, Thamboo A, Choby G, Ma Y, Zhou B, Hwang PH (2019). Outcomes of sinonasal inverted papilloma resection by surgical approach: an updated systematic review and meta-analysis. Int Forum Allergy Rhinol.

[23] Choi WR, Lee BJ, Kim JH (2019). Long-term outcome following resection of sinonasal inverted papillomas: a single surgeon’s experience in 127 patients. Clin Otolaryngol.

[24] Philpott CM, Dharamsi A, Witheford M, Javer AR (2010). Endoscopic management of inverted papillomas: long-term results—the St Paul’s Sinus Centre experience. Rhinology.

[25] Lombardi D, Tomenzoli D, Buttà L (2011). Limitations and complications of endoscopic surgery for treatment for sinonasal inverted papilloma: a reassessment after 212 cases. Head Neck.

[26] Smith O, Gullane PJ (1987). Inverting papilloma of the nose: analysis of 48 patients. J Otolaryngol.

[27] Busquets J, Hwang P (2006). Endoscopic resection of sinonasal inverted papilloma: a meta-analysis. Otolaryngol Head Neck Surg.

[28] Mirza S, Bradley PJ, Acharya A, Stacey M, Jones NS (2007). Sinonasal inverted papillomas: recurrence, and synchronous and metachronous malignancy. J Laryngol Otol.

[29] Walgama E, Ahn C, Batra PS (2012). Surgical management of frontal sinus inverted papilloma: a systematic review. Laryngoscope.

[30] Sham CL, van Hasselt CA, Chow SMW (2020). Frontal inverted papillomas: a 25-year study. Laryngoscope.

[31] Healy DY, Chhabra N, Metson R, Holbrook EH, Gray ST (2016). Surgical risk factors for recurrence of inverted papilloma. Laryngoscope.

[32] Lund VJ, Stammberger H, Nicolai P (2010). European position paper on endoscopic management of tumours of the nose, paranasal sinuses and skull base. Rhinol Suppl.

[33] Tong CCL, Patel NN, Maina IW (2019). Inverted papilloma with multifocal attachment is associated with increased recurrence. Int Forum Allergy Rhinol.

[34] Sbrana MF, Borges RFR, Pinna FDR, Neto DB, Voegels RL (2021). Sinonasal inverted papilloma: rate of recurrence and malignant transformation in 44 operated patients. Braz J Otorhinolaryngol.

[35] Gras-Cabrerizo JR, Montserrat-Gili JR, Massegur-Solench H, León-Vintró X, De Juan J, Fabra-Llopis JM (2010). Management of sinonasal inverted papillomas and comparison of classification staging systems. Am J Rhinol Allergy.

[36] Lisan Q, Moya-Plana A, Bonfils P (2017). Association of Krouse classification for sinonasal inverted papilloma with recurrence: a systematic review and meta-analysis. JAMA Otolaryngol Head Neck Surg.

[37] Derousseau T, Manjunath L, Harrow B, Zhang S, Batra PS (2015). Long-term changes in quality of life after endoscopic resection of sinonasal and skull-base tumors. Int Forum Allergy Rhinol.

[38] Deckard NA, Harrow BR, Barnett SL, Batra PS (2015). Comparative analysis of quality-of-life metrics after endoscopic surgery for sinonasal neoplasms. Am J Rhinol Allergy.

[39] van Samkar A, Georgalas C (2016). Long-term quality of life after endoscopic removal of sinonasal inverted papillomas: a 6-year cohort analysis in a tertiary academic hospital. Eur Arch Otorhinolaryngol.

[40] Gillett S, Hopkins C, Slack R, Browne JP (2009). A pilot study of the SNOT 22 score in adults with no sinonasal disease. Clin Otolaryngol.

[41] Bertazzoni G, Accorona R, Schreiber A (2017). Postoperative long-term morbidity of extended endoscopic maxillectomy for inverted papilloma. Rhinol J.

[42] Lin YH, Chen WC (2020). Clinical outcome of endonasal endoscopic prelacrimal approach in managing different maxillary pathologies. PeerJ.

[43] Jiang XD, Dong QZ, Li SL, Huang TQ, Zhang NK (2017). Endoscopic surgery of a sinonasal inverted papilloma: surgical strategy, follow-up, and recurrence rate. Am J Rhinol Allergy.

[44] Mohan M, Jagannathan N (2014). Oral field cancerization: an update on current concepts. Oncol Rev.

